# Engineered EryF hydroxylase improving heterologous polyketide erythronolide B production in *Escherichia coli*

**DOI:** 10.1111/1751-7915.14000

**Published:** 2022-02-17

**Authors:** Zhifeng Liu, Jianlin Xu, Haili Liu, Yong Wang

**Affiliations:** ^1^ CAS‐Key Laboratory of Synthetic Biology CAS Center for Excellence in Molecular Plant Sciences Institute of Plant Physiology and Ecology Chinese Academy of Sciences Shanghai 200032 China; ^2^ University of Chinese Academy of Sciences Beijing 100039 China; ^3^ State Key Laboratory of Bioreactor Engineering East China University of Science and Technology Shanghai 200237 China

## Abstract

In the last two decades, the production of complex polyketides such as erythromycin and its precursor 6‐deoxyerythronolide B (6‐dEB) was demonstrated feasible in *Escherichia coli*. Although the heterologous production of polyketide skeleton 6‐dEB has reached 210 mg l^−1^ in *E. coli*, the yield of its post‐modification products erythromycins remains to be improved. Cytochrome P450EryF catalyses the C6 hydroxylation of 6‐dEB to form erythronolide B (EB), which is the initial rate‐limiting modification in a multi‐step pathway to convert 6‐dEB into erythromycin. Here, we engineered hydroxylase EryF to improve the production of heterologous polyketide EB in *E. coli*. By comparative analysis of various versions of P450EryFs, we confirmed the optimal SaEryF for the biosynthesis of EB. Further mutation of SaEryF based on the crystal structure of SaEryF and homology modelling of AcEryF and AeEryF afforded the enhancement of EB production. The designed mutant of SaEryF, I379V, achieved the yield of 131 mg l^−1^ EB, which was fourfold to that produced by wild‐type SaEryF. Moreover, the combined mutagenesis of multiple residues led to further boost the EB concentration by another 41%, which laid the foundation for efficient heterologous biosynthesis of erythromycin or other complex polyketides.

## Graphical abstract

We engineered hydroxylase EryF to improve the production of heterologous polyketide EB in *E. coli*. Mutation of SaEryF based on the crystal structure of SaEryF and homology modeling of AcEryF and AeEryF afforded the enhancement of EB production. The designed mutant of SaEryF, I379V, achieved the yield of 131 mg l^−1^ EB, which was 4‐fold to that produced by wild‐type SaEryF. Moreover, the combined mutagenesis of multiple residues led to further boost the EB concentration by another 41%, which laid the foundation for efficient heterologous biosynthesis of erythromycin or other complex polyketides.

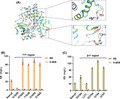

## Introduction

**Fig. 1:**
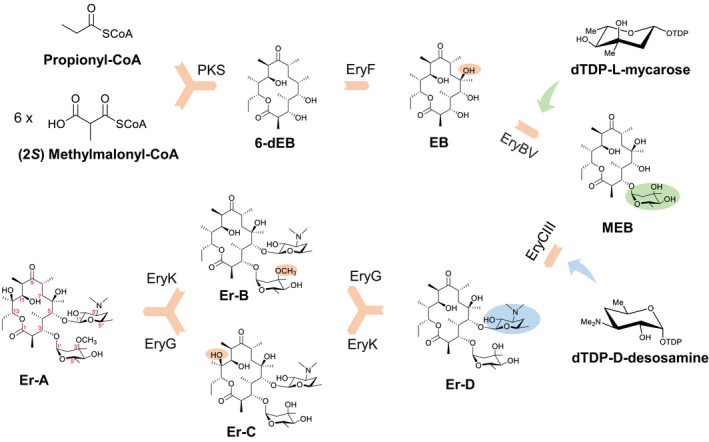
Biosynthetic pathway of erythromycin. 6‐dEB, 6‐deoxyerythronolide B; EB, erythronolide B; MEB, 3‐*O*‐α‐mycarosylerythronolide B; Er‐D, erythromycin D; Er‐B, erythromycin B; Er‐C, erythromycin C; Er‐A, erythromycin A. PKS, polyketide synthetase; EryF, C6‐hydroxylase; EryBV, l‐mycarosyltransferase; EryCIII, d‐desosaminyltransferase; EryG, C3′‐*O*‐methyltransferase; EryK, C12‐hydroxylase.

Polyketides are a large class of structurally diverse natural products derived from bacteria, fungi and plants, exhibiting a broad spectrum of bioactivities such as antibacterial, antifungal, antiviral, anticancer and immunosuppressive activities, some of which have been used for the treatment of diseases in humans and animals (Dinos, 2017). The macrolide antibiotic erythromycin is a typical member of the polyketide family. Due to its remarkable inhibitory effect on pathogenic bacteria, the biosynthesis of erythromycin has been studied in depth, which involves the formation of polyketide skeleton 6‐deoxyerythronolide B (6‐dEB) and its diversified modifications (Rawlings, 2001). Firstly, one propionyl‐CoA and six (2*S*)‐methylmalonyl‐CoAs are assembled into 14‐membered macrolide 6‐dEB by the 6‐deoxyerythronolide B synthetase. The initial reaction of modification involved in the conversion of 6‐dEB to erythronolide B (EB) is catalysed by the cytochrome P450EryF. The resultant EB is successively decorated with structurally diverse TDP‐L‐mycarose and TDP‐D‐desosamine by glycosyltransferases EryBV and EryCIII, leading to the generation of erythromycin D. The erythromycin D is further hydroxylated at the C12 and methylated at the C3′ under the sequential action of enzymes EryG and EryK, resulting in the production of erythromycin A (Fig. 1).

Since the erythromycin‐producing microorganisms are mainly complex and slow‐growing soil actinomycetes that make their production and isolation difficult, the culture and genetic manipulation superiority of the well‐characterized *Escherichia coli* when it is employed for the production of erythromycin is well established (Pfeifer and Khosla, 2001; Yuzawa *et al*., 2012). The production of polyketide skeleton (6‐dEB) has been significantly facilitated to 210 mg l^−1^ in *E. coli* via various engineering strategies including the optimization of precursor supply (Dayem *et al*., 2002; Murli *et al*., 2003; Zhang *et al*., 2010a; Boghigian *et al*., 2011; Vandova *et al*., 2017); bioprocess culture at high cell density (Lau *et al*., 2004); transporter and cofactor engineering (Wang *et al*., 2007; Yang *et al*., 2015); systematic metabolic engineering (Meng *et al*., 2016) and reconstruction of Wood‐Werkman cycle (Gonzalez‐Garcia *et al*., 2020a,2020b). Nevertheless, it is still challenging to divert 6‐dEB to erythromycin and acquire the final product erythromycin A with a high titre. The expression of 17 genes derived from *Micromonospora megalomicea* encoding two monosaccharide biosynthetic and tailoring enzymes enabled the 6‐dEB‐producing *E*. *coil* to produce erythromycins C and D with titres of 0.4 and 0.5 mg l^−1^, respectively (Peirú *et al*., 2005). When introducing the erythromycin gene cluster into *E. coil*, the complete biosynthesis of the most bioactive erythromycin A was accomplished and its yield reached 0.6 mg l^−1^ (Zhang *et al*., 2010b). Subsequently, 1.2 mg l^−1^ erythromycin A was achieved through redesigning the plasmids for erythromycin formation (Jiang *et al*., 2013; Fang *et al*., 2018). The possible reason that the titre of erythromycin in *E. coil* is low might be attributed to the inefficiency and promiscuity of certain enzymes accountable for decoration of 6‐dEB, leading to metabolic flux diversion and by‐products formation. It has been reported that inactivating the gene encoding P450EryF resulted in the formation of unwanted products such as the erythromycin derivatives lacking the C6 hydroxyl group (Weber *et al*., 1991). Moreover, the *in vitro* enzyme assay found that promiscuous glycosyltransferase EryBV could utilize alternative substrates 6‐dEB to produce 3‐*O*‐*α*‐mycarosylerythronolide B (MEB) analogues (Zhang *et al*., 2007).

Given that 6*S*‐hydroxylation is the first step of adornment of 6‐dEB, we speculated that the catalytic activity of hydroxylase P450EryF might be the critical biosynthetic bottleneck that impeded titre improvement of erythromycin. Consequently, enhancing the catalytic activity of P450EryF would contribute to improving the yield of erythromycin in *E. coil*. The P450EryF, a special bacterial P450 monooxygenase, which lacked the highly conserved threonine residue, compared with the other P450s currently discovered. The crystal structures of EryF in complex with multiple ligands provided insight into the molecular mechanism that P450EryF facilitated proton transfer and then triggered scission of the O − O bond via a complex hydrogen‐bonding network, which was comprised of the C5‐OH of 6‐dEB, three water molecules, as well as four amino acid side chains (Cupp‐Vickery and Poulos, 1995; Cupp‐Vickery *et al*., 1996). Furthermore, the cooperativity of substrate binding was elucidated by the crystal structures of P450EryF bound with steroid compounds and azole‐based steroid hydroxylase inhibitors (Cupp‐Vickery and Poulos, 1997; Cupp‐Vickery *et al*., 2000, 2001). Based on these structures, extensive investigations that engineering of P450EryF aimed to explore the structure–function relationships between P450EryF and steroid substrates were conducted (Xiang *et al*., 2000; Khan *et al*., 2002; Nagano *et al*., 2005). However, the effect of various P450EryF active‐site residues that are correlated to natural substrate binding and oxidation has been underexplored.

In this study, we first biosynthesized EB in *E. coli* and then achieved the improvement of EB titre through engineering of P450EryF. According to the kinetic parameters and EB fermentative production of three P450s, the optimal SaEryF was screened. The SaEryF was further engineered on the basis of its crystal structure and homology modelling of AcEryF and AeEryF, which optimized the titre of EB to 131 mg l^−1^ in an engineered *E. coli*. Lastly, the triple mutant I379V_G165S_A74F was designed and used to further improve the EB titre by another 41%.

## Results

### Biosynthesis of erythronolide B in *E. coli*

**Fig. 2:**
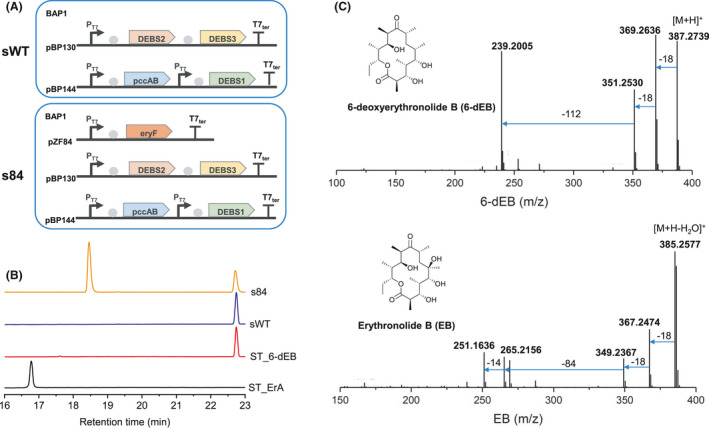
Biosynthesis of polyketide substrates erythronolide B (EB) in *E. coli*. A. The starting strain sWT and the EB producing strain s84. DEBS1, DEBS2 and DEBS3, the 6‐deoxyerythronolide B (6‐dEB) synthase. pccAB, propionyl‐CoA carboxylase. EryF, C6‐hydroxylase. B. The HPLC analysis of the fermentation product of sWT and s84. C. The LC‐MS/MS fragments of 6‐dEB and EB.

To establish the heterologous biosynthetic pathway of EB in *E. coli*, the previously reported BAP1 harbouring pBP130 and pBP144 for the production of 6‐dEB served as the starting strain (sWT) (Pfeifer *et al*., 2001). *SaEryF*, P450EryF gene from *Saccharopolyspora erythraea*, was synthesized with optimized codons and assembled into plasmid pCDFDuet‐1 under T7 promoter to generate pZF84 (Fig. 2A), which was transferred into sWT to create the recombinant strain s84. Two obvious peaks were detected in s84 broth after 120 h fermentation (Fig. 2B). The minor peak with a retention time (*R*_t_) = 22.7 min was determined as 6‐dEB in comparison with the standard, while the major peak with a retention time (*R*_t_ = 18.5 min) between erythromycin (*R*_t_ = 16.7 min) and 6‐dEB (*R*_t_ = 22.7 min) was predicted to be EB. To prove our deduction, the fermentation broth of s84 was extracted and then subjected to HPLC‐MS/MS analysis. Expectedly, two characteristic ion peaks *m*/*z* 385.2577 ([M + H−H_2_O]^+^, C_21_H_38_O_7_, calcd for 385.2584) and *m*/*z* 387.2739 ([M + H]^+^, C_21_H_38_O_6_, calcd for 387.2741) were observed in mass profiles, which suggested the presence of EB and 6‐dEB (Fig. 2C). After 3.6L‐scale fermentation and purification, 70 mg EB and 50 mg 6‐dEB with 95% purity were obtained, as evidenced by NMR spectra (Figs S1‐S4). The standard curves of 6‐dEB and EB were determined and used in the subsequent quantitative analysis (Figs S5 and S6).

### Screening EryF for improving the heterologous production of EB in *E. coli*

To increase the supply and availability of EB, we attempted to screen P450EryFs derived from different erythromycin‐producing strains to identify the most effective hydroxylase for EB biosynthesis (Brikun *et al*., 2004; Chen *et al*., 2014; Harrell and Miller, 2016). Three known P450EryF genes: *SaEryF* (from *S. erythraea*), *AcEryF* (from *Actinopolyspora erythraea*) and *AeEryF* (from *Aeromicrobium erythreum*) were synthesized with optimized codons and constructed into pCDFDuet‐1 with N‐terminal His‐tag. Three plasmids pZF71‐pZF73 were constructed and transformed into *E. coli* BL21 (DE3) for protein expression. Proteins (SaEryF, AcEryF and AeEryF) were purified with Ni^2+^‐NTA column and confirmed with SDS‐PAGE (Fig. S7), which were used for the subsequent enzymatic activity on C6 hydroxylation with 6‐dEB as substrate. The affinity and catalytic efficiency of each EryF were quantified by changing the concentration of 6‐dEB (Table 1). The *K*_m_ value of SaEryF was determined to be 13.7 μM, whereas the *K*_m_ values of AcEryF and AeEryF were 17.4 μM and 19.4 μM, respectively. In terms of *k*_cat_, there is no conspicuous difference among the aforementioned P450EryFs. The *k*_cat_/*K*_m_ values of SaEryF, AcEryF and AeEryF enzymes implied that SaEryF exhibited better catalytic property for the substrate 6‐dEB than AcEryF and AeEryF.

**Table 1:** Kinetic parameters of different EryF.

Kinetic parameter	SaEryF	AcEryF	AeEryF	I379V
*K*_m_ (μM)	13.66	17.43	19.42	0.2016
*k*_cat_ (min^−1^)	0.4315	0.4365	0.4514	0.6909
V_max_ (μM min^−1^)	4.315	4.365	4.514	6.909
*k*_cat_/*K*_m_ (μM^−1^ min^−1^)	0.031	0.025	0.023	3.427

**Fig. 3:**
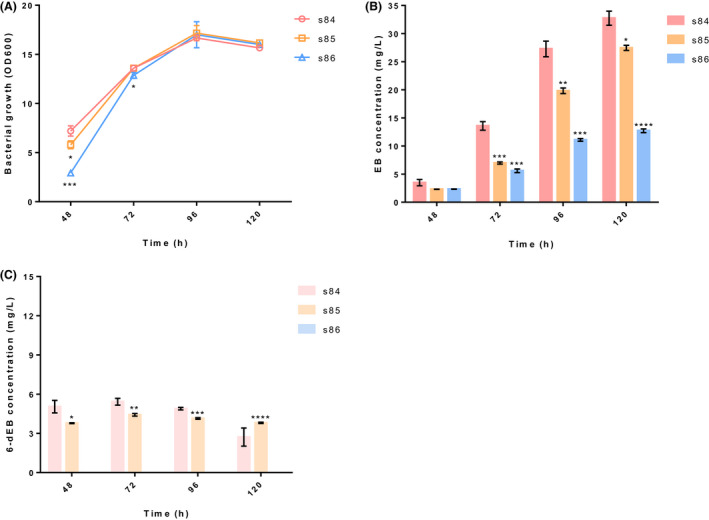
Comparison of the activities of SaEryF, AcEryF and AeEryF by flask fermentation in *E. coli*. A. The growth curve of s84, s85 and s86 in 120 h fermentation. B. The titre of EB in s84, s85 and s86. C. The titre of 6‐dEB in s84, s85 and s86. Data shown are means ± standard deviations calculated from triplicate individual experiments. Error bars show standard deviations. Statistical analysis was performed by a two‐tailed Student’s *t*‐test. *P < 0.05, **P < 0.01, ***P < 0.001 and ****P < 0.0001 vs. the s84 strain.

To compare the ability of SaEryF, AcEryF and AeEryF to catalyse 6‐dEB to EB *in vivo*, EB biosynthetic strains s85 and s86, which were constructed individually through the introduction of pZF85 (AcEryF‐expressing plasmid) and pZF86 (AeEryF‐expressing plasmid) into sWT, together with s84, were cultivated in shake flask for 120 h. Despite the fact that s84 grew faster than s85 and s86 within 72 h, all strains reached the same *OD*_600_ value at 120 h (Fig. 3A). The s84 afforded the highest EB production with a peak titre of 32.7 mg l^−1^ at 120 h, which is 1.2‐fold and 2.6‐fold increase to those produced by s85 (27.5 mg l^−1^) and s86 (12.7 mg l^−1^), respectively (Fig. 3B). Interestingly, there is no accumulations of 6‐dEB in s86, while the residue of 6‐dEB in s84 and s85 was 2.7 mg l^−1^ and 3.8 mg l^−1^, respectively (Fig. 3C). Our results revealed that SaEryF was able to achieve the highest yield of EB, which was consistent with the enzymatic activity *in vitro*.

### Effects of mutation of substrate‐binding pocket residues on EB titre

**Fig. 4:**
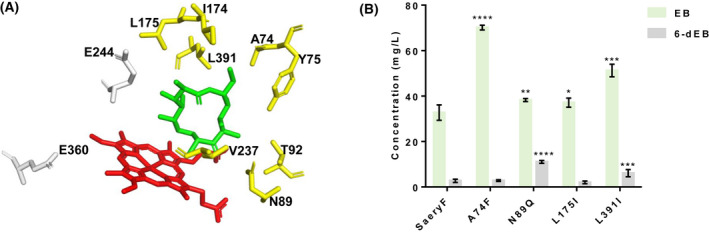
Effects of mutation of substrate‐binding pocket residues on EB titre. A. The substrate‐binding pocket of SaEryF (PDB: 1JIO). Red, heme; green, substrate 6‐dEB; yellow, residues involved in substrate‐binding; and grey, residues involved in hydrogen‐bonding network. B. The titre of EB and 6‐dEB of mutants A74F, N89Q, L175I and L391I. The data are presented as the means ± standard deviations calculated from three independent experiments. Error bars show standard deviations. Statistical analysis was performed by a two‐tailed Student’s *t*‐test. *P < 0.05, **P < 0.01, ***P < 0.001 and ****P < 0.0001 vs. the wild‐type SaEryF.

With the great efforts have made in engineering to improve the P450s catalytic efficiency that overcomes the inherent limitations of native enzymes (Li *et al*., 2020), we shifted our focus to probe the effect of mutation of residues involving the hydrogen‐bonding network and substrate recognition of SaEryF (Fig. 4A). Since the hydrogen‐bonding network could provide proton shuttle pathway for the hydroxylation process of 6‐dEB (Cupp‐Vickery and Poulos, 1995), fine‐tuning the residues involved in the hydrogen‐bonding network might affect the catalytic property of SaEryF. Two residues Glu360 and Glu244 that were proposed to be critical for the formation of hydrogen‐bonding network have yet to be experimentally characterized thus far (Cupp‐Vickery *et al*., 1996; Sen and Thiel, 2014). To enhance the concentrate of EB, two single mutations E244D and E360D were designed with the consideration of the most similar amino acid and transformed into sWT for subsequent verification. *In vivo* fermentation was implemented instead of *in vitro* enzyme assay, because we proved that it was consistent with *in vitro* characterization. Unexpectedly, mutants E244D and E360D only retained 17% and 38% of the relative EB titre of the wild‐type SaEryF (Fig. S8), whereas the high titre of 6‐dEB (more than 35 mg l^−1^) was accumulated in these two mutants. These results revealed that mutation of Glu244 and Glu360 in the network of hydrogen bonds might pose a detrimental effect on the catalytic function of SaEryF.

We next turned to alter residues around the substrate‐binding pocket by site‐directed mutagenesis. In the crystal structure of SaEryF (PDB: 1JIO), substrate 6‐dEB was bound in the active site through the interaction of eight amino acids (Ala74, Tyr75, Asn89, Thr92, Ile174, Leu175, Val237 and Leu391) as depicted with yellow colour (Fig. 4A). The wild‐type SaEryF could generate 32.7 mg l^−1^ EB and 2.7 mg l^−1^ 6‐dEB. Replacement of Ala74 with phenylalanine exhibited an increased EB titre and reached 70 mg l^−1^ (Fig. 4B), a 2.1‐fold to that of wild‐type SaEryF, which suggested that the larger side chain of phenylalanine was capable of enhancing the interaction between enzyme and substrate. However, substitution of Tyr75 with phenylalanine had a slight effect on EB production (Fig. S8). Surprisingly, when Asn89 was replaced with Gln, both the yield of EB and 6‐dEB was increased to 38 mg l^−1^ and 11 mg l^−1^, which is 1.1‐fold and 4.1‐fold to those of wild‐type SaEryF (Fig. 4B). As Asn89 formed hydrogen bond with the keto group of 6‐dEB in SaEryF, the substitution of asparagine with glutamine resulted in the shorter distances between enzyme and substrate, which probably strengthened the binding of substrate. Instead, T92S mutant significantly decreased the hydroxylation product (Fig. S8). Four hydrophobic residues (Ile174, Leu175, Val237 and Leu391) would be critical to shape the hydrophobic environment and influence the substrate binding. Therefore, we speculated that mutating these residues might result in a change in the catalysis property of SaEryF. Eight mutants were created and transformed into sWT for the use of fermentation. The shake flask fermentation results implied that the overall catalytic activities of six variants (I174L, I174V, L175V, V237L, V237I and L391V) declined to 22%–68% of the native SaEryF (Fig. S8), whereas mutants L175I and L391I showed a 1.1‐fold and 1.6‐fold increase in EB, respectively (Fig. 4B). These results explained from the perspective of structure that replacing Leu with Ile led to the shift of the space restriction, which helped to anchor the substrate in the catalytic centre and facilitate the catalytic efficiency of EryF. The negative effect on the EB titre of mutants I174L, I174V, L175V, V237L, V237I and L391V might result from the broken hydrophobic interaction between the protein and the substrate 6‐dEB (Cupp‐Vickery and Poulos, 1997). Additionally, the accumulation of 6‐dEB (6.1 mg l^−1^) was also detected in L391I, which is 2.3‐fold to that produced by wild‐type SaEryF. Taken together, modulating the substrate‐binding pocket could increase the final concentration of EB.

### Engineering SaEryF based on homology modelling

**Fig. 5:**
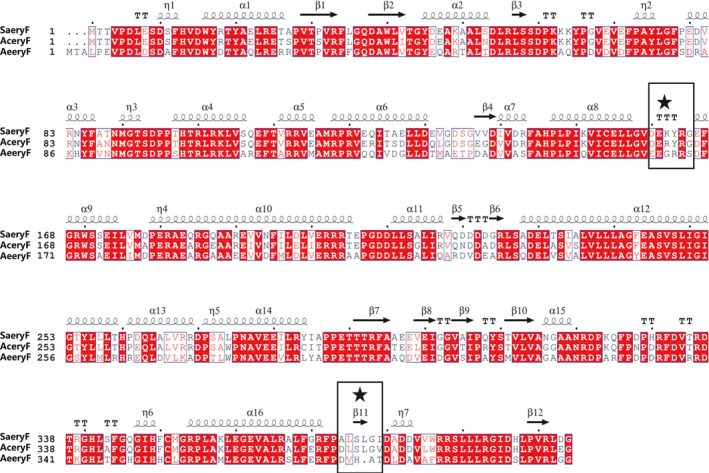
Sequence alignment of EryF enzymes with secondary structure indications. The EryF enzymes were derived from *S. erythraea* (SaEryF), *A. erythraea* (AcEryF) and *A. erythreum* (AeEryF). Black boxes indicate the TTT and β11 region. The red background shows sequence identity. Red letters show sequence similarity. The alignment was performed with ClustalW, and the figure was made with ESPript 3.

**Fig. 6:**
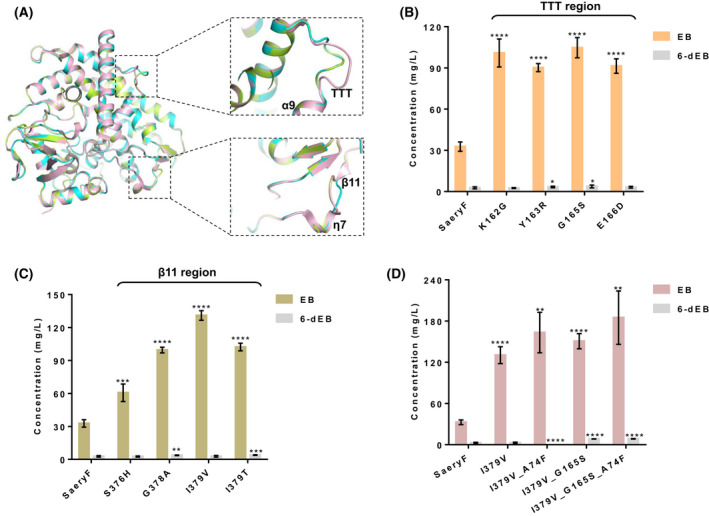
Engineering SaEryF based on homology model. A. Superposing the homology model of AcEryF and AeEryF with SaEryF. Pink, SaEryF (PDB:1JIO); limon, the constructed model of AcEryF; cyan, the constructed model of AeEryF. B. The titre of EB and 6‐dEB of mutants derived from residues in TTT regions. C. The titre of EB and 6‐dEB of mutants based on amino acids in β11 region. D. The titre of EB and 6‐dEB of double and triple mutants. The data shown are means ± standard deviations calculated from triplicate individual experiments. Error bars show standard deviations. Statistical analysis was performed by a two‐tailed Student’s *t*‐test. *P < 0.05, **P < 0.01, ***P < 0.001 and ****P < 0.0001 vs. the wild‐type SaEryF.

To further enhance the activity of SaEryF, we attempted to engineer SaEryF under the guidance of sequence alignment and homology modelling. The models of AcEryF and AeEryF were established using the crystal structure of 6‐dEB‐SaEryF complex (PDB: 1JIO) as a template. The amino acid sequence of the template protein shared approximately 88% identity to that of AcEryF and 69% identity to that of AeEryF (Fig. 5). Overlay of the crystal structure of SaEryF with the models of AcEryF and AeEryF showed that the models of AcEryF and AeEryF exhibited a high resemblance with the crystal structure of SaEryF, except for subtle differences in two regions TTT and β11. The TTT region links to *α*9 helix, which acts as a cap to close the entrance of the substrate‐binding pocket, and the β11 region is adjacent to *η*7 helix, which extends to the substrate‐binding pocket (Fig. 6A). Thus, we hypothesized that mutating these two regions would affect the activity of SaEryF.

To confirm the hypothesis, eight amino acids in these two regions were selected for mutation on the basis of sequence alignment. First, five mutants were devised based on the four residues in the less conserved TTT region (Fig. 5). A lysine residue (Lys162) in SaEryF was replaced with arginine in AcEryF and glycine in AeEryF, respectively, which led to the creation of mutants K162R and K162G. Similarly, mutants Y163R and G165S were generated via substituting Tyr163 and Gly165 in SaEryF with arginine and serine in AeEryF, respectively. Besides, replacing the Glu166 in SaEryF with aspartic acid in AcEryF and AeEryF created mutant E166D. Then, the strain sWT was used to screen for EB concentration changes *in vivo* by the mutations in TTT region. The EB production of K162R was reduced by 80% (Fig. S9), while K162G and G165S presented a twofold increase in the level of EB. Y163R and E166D exhibited 2.8‐fold the relative titre of EB of wild‐type SaEryF (Fig. 6B). This agrees with our hypothesis that the TTT region is important in regulating the entrance of substrate‐binding pocket.

Using a similar residue substitution strategy, mutants S376H, L377_ (the deletion of Leu377), G378A, I379V and I379T were generated according to the four residues in the β11 region. Except for the L377_ (Fig. S9), the other four mutants were able to boost EB production, among which I379V achieved the yield of 131 mg l^−1^, a fourfold to that of the wild‐type SaEryF (32.7 mg l^−1^) (Fig. 6C). It seems that the shorter β11 region redirects the position of the *η*7 helix, which promotes the substrate binding and recognition to a certain degree. The total intracellular protein concentrations of SaEryF mutants whose EB titre was dramatically enhanced were quantified by sodium dodecyl sulphate–polyacrylamide gel electrophoresis (SDS‐PAGE). As shown in Fig. S10, almost all selected mutations of SaEryF did not affect the protein expression, which indicated that the improvement of EB production was attributed to the effect of kinetic property instead of the change of protein expression. Further measurement of the kinetic parameters of mutant I379V showed that the *K*_m_ value and *k*_cat_/*K*_m_ ratio of mutant I379V was ˂ 2% and 110‐fold higher, respectively, compared with wild‐type SaEryF (Table 1). Taken together, the mutations in TTT and β11 region significantly improved the catalytic efficiency of SaEryF.

To further enhance the concentration of EB, the most beneficial mutations in substrate‐binding pocket and TTT region, namely A74F and G165S, were selected and combined with mutant I379V (Fig. 6D). The EB concentration of double mutants I379V_G165S and I379V_A74F was 150.6 and 163.2 mg l^−1^, respectively, which is a 15% and 25% increase to that produced by mutant I379V (131.0 mg l^−1^). Notably, when mutations A74F and G165S were simultaneously introduced to the I379V mutant, this triple mutant produced the highest titre of EB of 184.8 mg l^−1^, which is 1.4‐fold to that produced by mutant I379V and 5.6‐fold to that of wild‐type SaEryF.

## Discussion

The highly efficient biosynthesis of natural small molecules in heterologous hosts remains an overwhelming challenge in the field of metabolic engineering. One possible reason is that natural enzymes tend to function inefficiently in heterologous hosts and are difficult to support a high pathway flux (Keasling, 2010; Jeschek *et al*., 2017). Enzyme engineering is widely applied to overcome the bottlenecks of pathway enzymes, attributable to the improved metabolic flux towards targeted metabolite production (Eriksen *et al*., 2014). For example, the intrinsic promiscuity and inefficiency of carotenoid cleavage dioxygenase 1 (CCD1) limited the yield of *α*‐ionone in *E. coli*. Site‐directed mutagenesis of key rate‐limiting CCD1 was implemented in conjunction with enzyme fusion to boost the *α*‐ionone titre by >2.5‐fold (Chen *et al*., 2019).

In the present study, we successfully promoted the production of EB to 131 mg l^−1^ by means of engineering of the key enzyme SaEryF under the guide of structure and homology modelling. We postulated that the significant enhancement of EB titre might attribute to the catalytic contribution of hydroxylase with the substantial improvement of the *K*_m_ and *k*_cat_ of I379V (Table 1). Despite the fact that the total amount of EB and 6‐dEB in wild‐type SaEryF is 35.4 mg l^‐1^, the best engineered strain achieved 184.8 mg l^−1^ EB, which indicated that the high catalytic efficiency of engineered enzyme supported high pathway metabolic flux towards EB production *in vivo* fermentation. Moreover, the simultaneous increase in both 6‐dEB and EB in variants Y163R, G165S, G378A and I379T also demonstrated that the metabolic flux channel to the biosynthesis of desired metabolites (Fig. 6B and C).

In summary, we engineered the rate‐limiting enzyme SaEryF under the guide of structure and homology modelling, which significantly improved the EB concentration in *E. coli*. It is worthy to note that the EB production of the triple mutant I379V_G165S_A74F reached 184.8 mg l^−1^, the highest yield of EB produced by *E. coli*, which is a 5.6‐fold increase to that of native SaeryF (32.7 mg l^−1^). Importantly, this research provides new sight into the catalytic property of SaeryF and establishes the basis for further titre improvement of erythromycin or other complex polyketides in *E. coli*.

## Experimental procedures

### Strains, plasmids and chemicals

*E. coli* DH10B was used for plasmid propagation and construction. *E. coli* BL21 (DE3) was used for protein expression. The vector pCDFDuet‐1 was used to express different EryFs. HEPES‐free acid used in fermentation was bought from Sangon (Shanghai, China). Authentic chemical standards were purchased from Sangon. All restriction enzymes and DNA ligases were bought from NEB (New England Biolabs, Beverly, MA, USA).

### Plasmid construction

The encoding sequences of SaEryF (CAM00071) from *S. erythraea*, AcEryF (AIS23778) from *Actinopolyspora erythraea* YIM90600 and AeEryF (ALX06070) from *Aeromicrobium erythreum* were synthesized by GenScript (Nanjing, China) with codon optimization for *E. coli* (Table S3) and individually cloned into pCDFDuet‐1 between the HindIII/NotI site for protein expression, and the resulting plasmids were named pZF71–pZF73 (Table 2). The coding sequence of ferredoxin‐NADP reductase (XM_022006538) from *Spinacia oleracea* was synthesized by GenScript (Nanjing, China) with codon optimization for *E. coli* (Table S3) and cloned into pET21a between NdeI/NotI to create pZF70 (Table 2).

**Table 2:** strains and plasmids used in this study.

Plasmid/strain	Description	Source
Plasmid		
pBP130	pET21c‐P_T7_‐*DEBS2*‐*DEBS3*‐T7ter	(Pfeifer *et al*., 2001)
pBP144	pET28a‐P_T7_‐*pccB*‐rbs‐*pccA*‐P_T7_‐*DEBS1*‐T7ter	(Pfeifer *et al*., 2001)
pZF70	pET21a‐P_T7_‐*FNR*‐6xHis‐T7ter	This study
pZF71	pCDFDuet‐P_T7_‐6xHis‐*SaEryF*‐T7ter	This study
pZF72	pCDFDuet‐P_T7_‐6xHis‐*AcEryF*‐T7ter	This study
pZF73	pCDFDuet‐P_T7_‐6xHis‐*AeEryF*‐T7ter	This study
pZF84	pCDFDuet‐P_T7‐_ *SaEryF*‐T7ter	This study
pZF85	pCDFDuet‐P_T7_‐*AcEryF*‐T7ter	This study
pZF86	pCDFDuet‐P_T7_‐*AeEryF*‐T7ter	This study
Strain		
BAP1	F‐ompT hsdSB (rB‐mB‐) gal dcm (DE3) prpRBCD::PT7‐sfp, PT7‐prpE	(Pfeifer *et al*., 2001)
sWT	BAP1 carrying pBP130, pBP144	This study
s84	BAP1 carrying pBP130, pBP144, pZF84	This study
s85	BAP1 carrying pBP130, pBP144, pZF85	This study
s86	BAP1 carrying pBP130, pBP144, pZF86	This study

For the heterologous biosynthesis of EB, the coding sequences of SaEryF, AcEryF and AeEryF were amplified from pZF71, pZF72 and pZF73 using primer_84F/R, primer_85F/R and primer_86F/R (Table S2) and inserted into the linearized pCDFDuet‐1 to generate pZF84‐pZF86 (Table 2).

Sequences verified plasmids of SaEryF mutation and corresponding strains for EB fermentation are provided in Table S1. For the construction of SaEryF mutants, PCR amplification was performed using plasmid pZF84 as template and site‐mutagenesis primers (Table S2). The obtained PCR fragments were further purified by gel electrophoresis and transformed into *E. coli* DH10B.

### Protein expression and purification

The expression plasmids (pZF70‐pZF73) were transformed into *E. coli* BL21 (DE3) for protein overexpression. The transformants were selected on LB plates supplemented with 50 μg ml^‐1^ spectinomycin at 37°C overnight. Single colony cultivated in liquid LB (supplemented with 50 μg ml^‐1^ spectinomycin) overnight and then inoculated (1 : 100) into 1 litre LB with 50 μg ml^‐1^ spectinomycin and grew at 37°C in an MQD‐S2R shaker at 200 rpm until *OD*_600_ reached 0.6. A final concentration of 0.5 mM isopropyl *β*‐D‐thiogalactopyranoside (IPTG) was added to induce protein expression. The cultures were further grown at 22°C for 20 h. Cells were harvested and resuspended in buffer C (100 mM potassium phosphate, 10% v/v glycerol, pH 7.4) supplemented with 1 mM phenylmethylsulfonyl fluoride, 1 mM EDTA, 1 mM DTT, 25 mM MgCl_2_ and 5 μg ml^‐1^ DNase I. Cells were crashed by C3 high‐pressure cell disruptor (Sunnybay Biotech, Ottawa, Canada), and the lysate was centrifuged at 10 000 rpm for 120 min (Centrifuge 5804R; Eppendorf, Hamburg, Germany). The supernatant was loaded onto a Ni^2+^‐NTA affinity column (Qiagen, Germantown, Maryland) and incubated at 4°C for 1 h, which was washed with buffer C supplemented with 25 mM imidazole and 1 mM DTT. The target proteins were eluted by buffer C supplemented with 250 mM imidazole and 1 mM DTT. The collected target proteins were concentrated into 1 ml using an Amicon Ultra‐15 Centrifugal Filter Unit (EMD Millipore). Protein concentration was determined using the Bradford assay (Sangon Biotech, Shanghai, China) in triplicate measurements.

### *In vitro* enzyme assay

A standard reaction (100 μl) consists of buffer C, 10 μg EryF protein, 5 mM spinach ferredoxin, 10 μg spinach ferredoxin‐NADP oxidoreductase (FNR), 10 mM glucose‐6‐phosphate, 2 U glucose‐6‐phosphate dehydrogenase, 2 mM NADPH and substrate 6‐dEB with concentration varying from 5 to 300 μM. The reaction was incubated at 30°C for 15 min and then extracted three times with an equal volume of ethyl acetate. The organic layer was collected and dried under vacuum, dissolved in 100 μl methanol and analysed by high‐performance liquid chromatography as described below. The enzyme kinetic parameters were calculated by quantification of the formation of EB. All data were presented as means ± SD from triplicate measurements. Michaelis–Menten curves were calculated by GraphPad Prism 7 (GraphPad Software, San Diego, CA, USA).

### Culture media and conditions of flask fermentation

Fermentation medium consists of Luria broth (LB), 15 g l^‐1^ glycerol and 100 mM 4‐(2‐hydroxyethyl)‐1‐piperazi‐neethanesuffonic acid (HEPES) buffer and was adjusted to pH 7.6 by NaOH before autoclaving. For the biosynthesis of EB, 100 μl of seed inoculum was inoculated into a 100 ml flask containing 10 ml fermentation medium supplemented with ampicillin (100 mg l^−1^), kanamycin (50 mg l^−1^) and spectinomycin (50 mg l^−1^) for propagation at 37°C. Isopropyl *β*‐D‐thiogalactopyranoside (IPTG) and sodium propionate were added at a final concentration of 0.5 and 5 mM when *OD*_600_ reached 0.4. Cell cultures were subsequently incubated at 22°C for 5 days.

### HPLC and LC‐MS/MS analytical methods

Samples were analysed by Dionex UltiMate 3000 HPLC analysis system (Thermo Scientific, Waltham, MA, USA) with ELSD detector (Alltech U3000; Agilent, Santa Clara, CA, USA) and a C18 column (SilGreen ODS column [φ 4.6 × 250 mm, S‐5 μm]; Greenherbs, Beijing, China) with a flow rate of 1 ml min^‐1^ at 30°C. Compounds were separated by acetonitrile (solvent A) and water (containing 50 mM ammonium formate, solvent B) under the following conditions: 0 min:100% B; 0–30 min: linear gradient increase to 95% A in 5% B; 30–31 min: linear gradient increase to100% B; 31–35 min: 100% B.

To obtain high purity of 6‐dEB and EB, collected samples were first separated by column chromatography over SiliaSphere C18 (50 µm; Silicycle, QuébecK, QC, Canada) and then purified by semi‐preparative HPLC (Dionex UltiMate 3000 Semi‐Preparative HPLC Systems; Thermo Scientific) with 40% acetonitrile in water (flow rate of 10 ml min^‐1^, detected at 205 nm) and a C18 column (SilGreen ODS column [φ 20 × 250 mm, 5 μm]; Greenherbs Co., Ltd., Beijing, China). ^1^H and ^13^C and 2D NMR spectra of 6‐dEB and EB were recorded by an Avance DRX 400 (500 MHz for ^1^H and 125 MHz for ^13^C) spectrometer (Bruker, Karlsruhe, Germany).

LC‐MS/MS was performed on Q Exactive hybrid quadrupole‐Orbitrap mass spectrometer (Thermo Scientific, Waltham, MA, USA) equipped with an Acquity UPLC BEH C18 column (φ 2.1 × 100 mm, 1.7 μm; Waters, Milford, MA, USA). The mobile phase was acetonitrile (A) and H_2_O with 0.1% formic acid (B). A linear gradient was set as follows: from 5 to 95% solvent A for 20 min; 95% solvent A for 5 min; and from 95 to 5% solvent A at 1 min. The flow rate was 0.3 ml min^‐1^, and the injection volume was 5 μl. The mass acquisition was performed in positive ionization mode with a full scan (100–1000).

### Homology modelling

Homology models of AcEryF and AeEryF were generated using the modelling server SWISS‐MODEL (https://swissmodel.expasy.org/) with the SaEryF structure (PDB: 1JIO) serving as a template (Cupp‐Vickery *et al*., 2001).

## Conflict of interest

The authors have no conflict of interest to declare.

## Supporting information


**Fig. S1**. ^1^H NMR spectrum (500 MHz, CD_3_OD) of 6‐deoxyerythronolide B.
**Fig. S2**. ^13^C NMR spectrum (125 MHz, CD_3_OD) of 6‐deoxyerythronolide B.
**Fig. S3**. ^1^H NMR spectrum (500 MHz, CD_3_OD) of erythronolide B.
**Fig. S4**. ^13^C NMR spectrum (125 MHz, CD_3_OD) of erythronolide B.
**Fig. S5**. Standard curve of 6‐deoxyerythronolide B.
**Fig. S6**. Standard curve of erythronolide B.
**Fig. S7**. SDS‐PAGE examination of the expression of purified protein. M represents protein marker; lane1, ferredoxin‐NADP oxidoreductase from *Spinacia oleracea*; lane 2, SaEryF, P450EryF from *S. erythraea*; lane 3, AcEryF, P450EryF from *A. erythraea*; lane 4, AeEryF, P450EryF from *A. erythreum*.
**Fig. S8**. Titre of EB and 6‐dEB of mutants derived from substrate‐binding pocket of SaEryF. The data shown are means ± standard deviations calculated from triplicate individual experiments. Error bars show standard deviations. Statistical analysis was performed by a two‐tailed Student’s *t*‐test. *P < 0.05, **P < 0.01, ***P < 0.001, and ****P < 0.0001 vs. the wild‐type SaEryF.
**Fig. S9**. Titre of EB and 6‐dEB of mutants K162R and L377_. L377_ means the deletion of amino acid Leu377. The data shown are means ± standard deviations calculated from triplicate individual experiments. Error bars show standard deviations. Statistical analysis was performed by a two‐tailed Student’s *t*‐test. *P < 0.05, **P < 0.01, ***P < 0.001, and ****P < 0.0001 vs. the wild‐type SaEryF.
**Fig. S10**. SDS‐PAGE examination of the expression of mutant protein. M represents protein marker; lane 1, control plasmid pCDFDuet‐1; lane 2, native SaEryF; lane 3, A74F; lane 4, N89Q; lane 5, L175I; lane 6, L391I; lane 7, K162G; lane 8, G165S; lane 9, I379V; and lane 10, I379T.
**Table S1**. Plasmids and strains for SaEryF mutation.
**Table S2**. Primers for plasmid construction.
**Table S3**. Synthesized DNA sequences in this study.

## References

[mbt214000-bib-0001] Boghigian, B.A. , Zhang, H. , and Pfeifer, B.A. (2011) Multi‐factorial engineering of heterologous polyketide production in *Escherichia coli* reveals complex pathway interactions. Biotechnol Bioeng 108: 1360–1371. 21337322 10.1002/bit.23069 PMC3076518

[mbt214000-bib-0002] Brikun, I.A. , Reeves, A.R. , Cernota, W.H. , Luu, M.B. , and Weber, J.M. (2004) The erythromycin biosynthetic gene cluster of *Aeromicrobium erythreum* . J Ind Microbiol Biotechnol 31: 335–344. 15257441 10.1007/s10295-004-0154-5

[mbt214000-bib-0003] Chen, D. , Feng, J. , Huang, L. , Zhang, Q. , Wu, J. , Zhu, X. , *et al*. (2014) Identification and characterization of a new erythromycin biosynthetic gene cluster in *Actinopolyspora erythraea* YIM90600, a novel erythronolide‐producing halophilic actinomycete isolated from salt field. PLoS One 9: e108129. 25250723 10.1371/journal.pone.0108129 PMC4176971

[mbt214000-bib-0004] Chen, X. , Shukal, S. , and Zhang, C. (2019) Integrating enzyme and metabolic engineering tools for enhanced α‐ionone production. J Agric Food Chem 67: 13451–13459. 31079451 10.1021/acs.jafc.9b00860

[mbt214000-bib-0005] Cupp‐Vickery, J. , Anderson, R. , and Hatziris, Z. (2000) Crystal structures of ligand complexes of P450eryF exhibiting homotropic cooperativity. Proc Natl Acad Sci USA 97: 3050–3055. 10716705 10.1073/pnas.050406897 PMC16190

[mbt214000-bib-0006] Cupp‐Vickery, J.R. , Garcia, C. , Hofacre, A. , and McGee‐Estrada, K. (2001) Ketoconazole‐induced conformational changes in the active site of cytochrome P450eryF. J Mol Biol 311: 101–110. 11469860 10.1006/jmbi.2001.4803

[mbt214000-bib-0007] Cupp‐Vickery, J.R. , Han, O. , Hutchinson, C.R. , and Poulos, T.L. (1996) Substrate‐assisted catalysis in cytochrome P450eryF. Nat Struct Biol 3: 632–637. 8673608 10.1038/nsb0796-632

[mbt214000-bib-0008] Cupp‐Vickery, J.R. , and Poulos, T.L. (1995) Structure of cytochrome P450eryF involved in erythromycin biosynthesis. Nat Struct Biol 2: 144–153. 7749919 10.1038/nsb0295-144

[mbt214000-bib-0009] Cupp‐Vickery, J.R. , and Poulos, T.L. (1997) Structure of cytochrome P450eryF: substrate, inhibitors, and model compounds bound in the active site. Steroids 62: 112–116. 9029724 10.1016/s0039-128x(96)00168-7

[mbt214000-bib-0010] Dayem, L.C. , Carney, J.R. , Santi, D.V. , Pfeifer, B.A. , Khosla, C. , and Kealey, J.T. (2002) Metabolic engineering of a methylmalonyl‐CoA mutase− epimerase pathway for complex polyketide biosynthesis in *Escherichia coli* . Biochemistry 41: 5193–5201. 11955068 10.1021/bi015593k

[mbt214000-bib-0011] Dinos, G.P. (2017) The macrolide antibiotic renaissance. Br J Pharmacol 174: 2967–2983. 28664582 10.1111/bph.13936 PMC5573421

[mbt214000-bib-0012] Eriksen, D.T. , Lian, J. , and Zhao, H. (2014) Protein design for pathway engineering. J Struct Biol 185: 234–242. 23558037 10.1016/j.jsb.2013.03.011 PMC3732524

[mbt214000-bib-0013] Fang, L. , Guell, M. , Church, G.M. , and Pfeifer, B.A. (2018) Heterologous erythromycin production across strain and plasmid construction. Biotechnol Prog 34: 271–276. 28960932 10.1002/btpr.2567 PMC5821549

[mbt214000-bib-0014] Gonzalez‐Garcia, R.A. , McCubbin, T. , Turner, M.S. , Nielsen, L.K. , and Marcellin, E. (2020a) Engineering *Escherichia coli* for propionic acid production through the Wood‐Werkman cycle. Biotechnol Bioeng 117: 167–183. 31556457 10.1002/bit.27182

[mbt214000-bib-0015] Gonzalez‐Garcia, R.A. , Nielsen, L.K. , and Marcellin, E. (2020b) Heterologous production of 6‐deoxyerythronolide B in *Escherichia coli* through the Wood Werkman Cycle. Metabolites 10: 228. 10.3390/metabo10060228 PMC7344785 32492827

[mbt214000-bib-0016] Harrell, E.A. , and Miller, E.S. (2016) Genome sequence of *Aeromicrobium erythreum* NRRL B‐3381, an erythromycin‐producing bacterium of the *Nocardioidaceae* . Genome Announc 4: e00300–e316. 27103725 10.1128/genomeA.00300-16 PMC4841140

[mbt214000-bib-0017] Jeschek, M. , Gerngross, D. , and Panke, S. (2017) Combinatorial pathway optimization for streamlined metabolic engineering. Curr Opin Biotechnol 47: 142–151. 28750202 10.1016/j.copbio.2017.06.014

[mbt214000-bib-0018] Jiang, M. , Fang, L. , and Pfeifer, B.A. (2013) Improved heterologous erythromycin A production through expression plasmid re‐design. Biotechnol Prog 29: 862–869. 23804312 10.1002/btpr.1759

[mbt214000-bib-0019] Keasling, J.D. (2010) Manufacturing molecules through metabolic engineering. Science 330: 1355–1358. 21127247 10.1126/science.1193990

[mbt214000-bib-0020] Khan, K.K. , He, Y.A. , He, Y.Q. , and Halpert, J.R. (2002) Site‐directed mutagenesis of cytochrome P450eryF: implications for substrate oxidation, cooperativity, and topology of the active site. Chem Res Toxicol 15: 843–853. 12067252 10.1021/tx025539k

[mbt214000-bib-0021] Lau, J. , Tran, C. , Licari, P. , and Galazzo, J. (2004) Development of a high cell‐density fed‐batch bioprocess for the heterologous production of 6‐deoxyerythronolide B in *Escherichia coli* . J Biotechnol 110: 95–103. 15099909 10.1016/j.jbiotec.2004.02.001

[mbt214000-bib-0022] Li, Z. , Jiang, Y. , Guengerich, F.P. , Ma, L. , Li, S. , and Zhang, W. (2020) Engineering cytochrome P450 enzyme systems for biomedical and biotechnological applications. J Biol Chem 295: 833–849. 31811088 10.1074/jbc.REV119.008758 PMC6970918

[mbt214000-bib-0023] Meng, H.L. , Xiong, Z.Q. , Song, S.J. , Wang, J. , and Wang, Y. (2016) Construction of polyketide overproducing *Escherichia coli* strains via synthetic antisense RNAs based on in silico fluxome analysis and comparative transcriptome analysis. Biotechnol J 11: 530–541. 26709503 10.1002/biot.201500351

[mbt214000-bib-0024] Murli, S. , Kennedy, J. , Dayem, L.C. , Carney, J.R. , and Kealey, J.T. (2003) Metabolic engineering of *Escherichia coli* for improved 6‐deoxyerythronolide B production. J Ind Microbiol Biotechnol 30: 500–509. 12898389 10.1007/s10295-003-0073-x

[mbt214000-bib-0025] Nagano, S. , Cupp‐Vickery, J.R. , and Poulos, T.L. (2005) Crystal structures of the ferrous dioxygen complex of wild‐type cytochrome P450eryF and its mutants, A245S and A245T: investigation of the proton transfer system in P450eryF. J Biol Chem 280: 22102–22107. 15824115 10.1074/jbc.M501732200

[mbt214000-bib-0026] Peirú, S. , Menzella, H.G. , Rodriguez, E. , Carney, J. , and Gramajo, H. (2005) Production of the potent antibacterial polyketide erythromycin C in *Escherichia coli* . Appl Environ Microbiol 71: 2539–2547. 15870344 10.1128/AEM.71.5.2539-2547.2005 PMC1087553

[mbt214000-bib-0027] Pfeifer, B.A. , Admiraal, S.J. , Gramajo, H. , Cane, D.E. , and Khosla, C. (2001) Biosynthesis of complex polyketides in a metabolically engineered strain of *E. coli* . Science 291: 1790–1792. 11230695 10.1126/science.1058092

[mbt214000-bib-0028] Pfeifer, B.A. , and Khosla, C. (2001) Biosynthesis of polyketides in heterologous hosts. Microbiol Mol Biol Rev 65: 106–118. 11238987 10.1128/MMBR.65.1.106-118.2001 PMC99020

[mbt214000-bib-0029] Rawlings, B.J. (2001) Type I polyketide biosynthesis in bacteria (Part A–erythromycin biosynthesis). Nat Prod Rep 18: 190–227. 11336289 10.1039/b009329g

[mbt214000-bib-0030] Sen, K. , and Thiel, W. (2014) Role of two alternate water networks in compound I formation in P450eryF. J Phys Chem B 118: 2810–2820. 24564366 10.1021/jp411272h

[mbt214000-bib-0031] Vandova, G.A. , O'Brien, R.V. , Lowry, B. , Robbins, T.F. , Fischer, C.R. , Davis, R.W. , *et al*. (2017) Heterologous expression of diverse propionyl‐CoA carboxylases affects polyketide production in *Escherichia coli* . J Antibiot (Tokyo) 70: 859–863. 28400575 10.1038/ja.2017.38 PMC5509990

[mbt214000-bib-0032] Wang, Y. , Boghigian, B.A. , and Pfeifer, B.A. (2007) Improving heterologous polyketide production in *Escherichia coli* by overexpression of an *S*‐adenosylmethionine synthetase gene. Appl Microbiol Biotechnol 77: 367–373. 17876579 10.1007/s00253-007-1172-9

[mbt214000-bib-0033] Weber, J.M. , Leung, J.O. , Swanson, S.J. , Idler, K.B. , and McAlpine, J.B. (1991) An erythromycin derivative produced by targeted gene disruption in *Saccharopolyspora erythraea* . Science 252: 114–117. 2011746 10.1126/science.2011746

[mbt214000-bib-0034] Xiang, H. , Tschirret‐Guth, R.A. , and Ortiz De Montellano, P.R. (2000) An A245T mutation conveys on cytochrome P450eryF the ability to oxidize alternative substrates. J Biol Chem 275: 35999–36006. 10956654 10.1074/jbc.M005811200

[mbt214000-bib-0035] Yang, J. , Xiong, Z.‐Q. , Song, S.‐J. , Wang, J.‐F. , Lv, H.‐J. , and Wang, Y. (2015) Improving heterologous polyketide production in *Escherichia coli* by transporter engineering. Appl Microbiol Biotechnol 99: 8691–8700. 26062534 10.1007/s00253-015-6718-7

[mbt214000-bib-0036] Yuzawa, S. , Kim, W. , Katz, L. , and Keasling, J.D. (2012) Heterologous production of polyketides by modular type I polyketide synthases in *Escherichia coli* . Curr Opin Biotechnol 23: 727–735. 22244790 10.1016/j.copbio.2011.12.029

[mbt214000-bib-0037] Zhang, C. , Fu, Q. , Albermann, C. , Li, L. , and Thorson, J.S. (2007) The in vitro characterization of the erythronolide mycarosyltransferase EryBV and its utility in macrolide diversification. ChemBioChem 8: 385–390. 17262863 10.1002/cbic.200600509

[mbt214000-bib-0038] Zhang, H. , Boghigian, B.A. , and Pfeifer, B.A. (2010a) Investigating the role of native propionyl‐CoA and methylmalonyl‐CoA metabolism on heterologous polyketide production in *Escherichia coli* . Biotechnol Bioeng 105: 567–573. 19806677 10.1002/bit.22560 PMC9896014

[mbt214000-bib-0039] Zhang, H. , Wang, Y. , Wu, J. , Skalina, K. , and Pfeifer, B.A. (2010b) Complete biosynthesis of erythromycin A and designed analogs using *E. coli* as a heterologous host. Chem Biol 17: 1232–1240. 21095573 10.1016/j.chembiol.2010.09.013

