# Supplementary material for: Engineered EryF hydroxylase improving heterologous polyketide erythronolide B production in Escherichia coli
Source: Microb Biotechnol. 2022 Feb 17;15(5):1598–609. doi: 10.1111/1751-7915.14000 (PMC9049603; doi:10.1111/1751-7915.14000)
Supplement: Supplementary file 1 — Fig. S1. 1H NMR spectrum (500 MHz, CD3OD) of 6‐deoxyerythronolide B. Fig. S2. 13C NMR spectrum (125 MHz, CD3OD) of 6‐deoxyerythronolide B. Fig. S3. 1H NMR spectrum (500 MHz, CD3OD) of erythronolide B. Fig. S4. 13C NMR spectrum (125 MHz, CD3OD) of erythronolide B. Fig. S5. Standard curve of 6‐deoxyerythronolide B. Fig. S6. Standard curve of erythronolide B. Fig. S7. SDS‐PAGE examination of the expression of purified protein. M represents protein marker; lane1, ferredoxin‐NADP oxidoreductase from Spinacia oleracea; lane 2, SaEryF, P450EryF from S. erythraea; lane 3, AcEryF, P450EryF from A. erythraea; lane 4, AeEryF, P450EryF from A. erythreum. Fig. S8. Titre of EB and 6‐dEB of mutants derived from substrate‐binding pocket of SaEryF. The data shown are means ± standard deviations calculated from triplicate individual experiments. Error bars show standard deviations. Statistical analysis was performed by a two‐tailed Student’s t‐test. *P < 0.05, **P < 0.01, ***P < 0.001, and ****P < 0.0001 vs. the wild‐type SaEryF. Fig. S9. Titre of EB and 6‐dEB of mutants K162R and L377_. L377_ means the deletion of amino acid Leu377. The data shown are means ± standard deviations calculated from triplicate individual experiments. Error bars show standard deviations. Statistical analysis was performed by a two‐tailed Student’s t‐test. *P < 0.05, **P < 0.01, ***P < 0.001, and ****P < 0.0001 vs. the wild‐type SaEryF. Fig. S10. SDS‐PAGE examination of the expression of mutant protein. M represents protein marker; lane 1, control plasmid pCDFDuet‐1; lane 2, native SaEryF; lane 3, A74F; lane 4, N89Q; lane 5, L175I; lane 6, L391I; lane 7, K162G; lane 8, G165S; lane 9, I379V; and lane 10, I379T. Table S1. Plasmids and strains for SaEryF mutation. Table S2. Primers for plasmid construction. Table S3. Synthesized DNA sequences in this study. [file MBT2-15-1598-s001.pdf]

Supporting Information

Engineered EryF hydroxylase improving heterologous polyketide erythronolide B production in *Escherichia coli*

Zhifeng Liu [1,2,†] Jianlin Xu [1,2,3,†] Haili Liu [1] and Yong Wang [1,*]

[1]CAS-Key Laboratory of Synthetic Biology, CAS Center for Excellence in Molecular Plant Sciences, Institute of Plant Physiology and Ecology, Chinese Academy of Sciences, Shanghai 200032, China

[2]University of Chinese Academy of Sciences, Beijing 100039, China

[3]State Key Laboratory of Bioreactor Engineering, East China University of Science and Technology, Shanghai 200237, China

*Corresponding author

E-mail: yongwang@cemps.ac.cn

Tel/fax: +86-21-54924295

†These authors contributed equally to this work.

Supplemental Figures

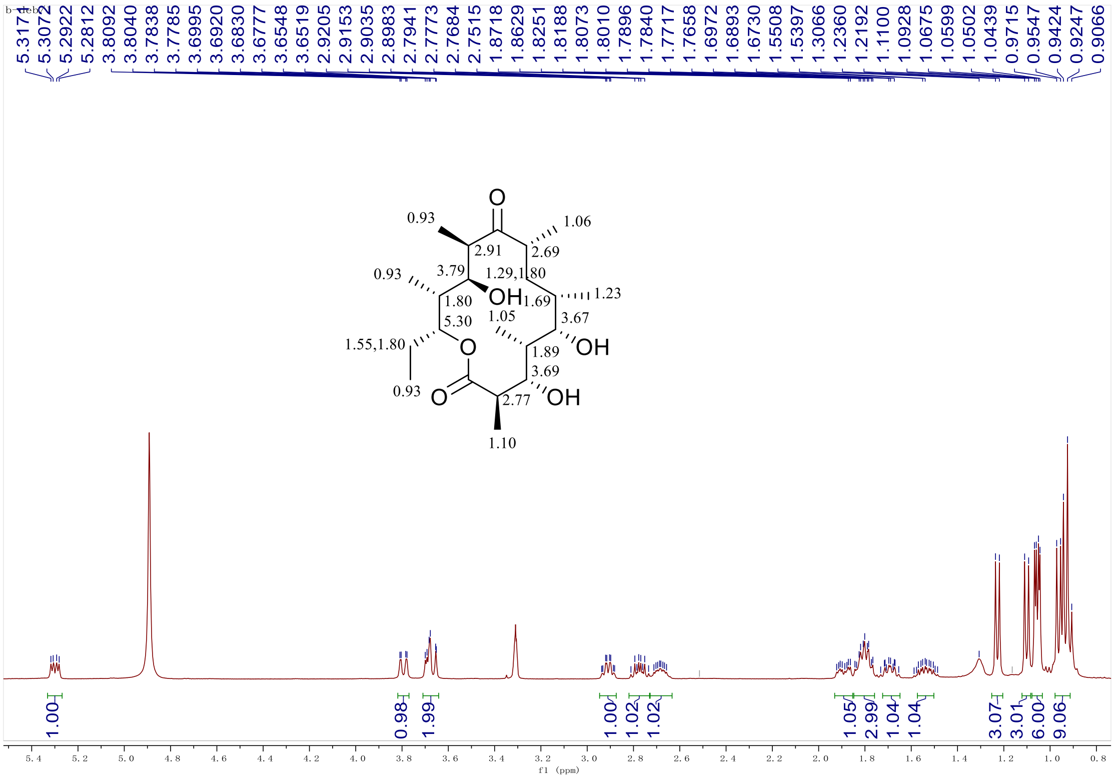

Fig. S1. ^{1}H NMR spectrum (500 MHz, CD_3OD) of 6-deoxyerythronolide B.

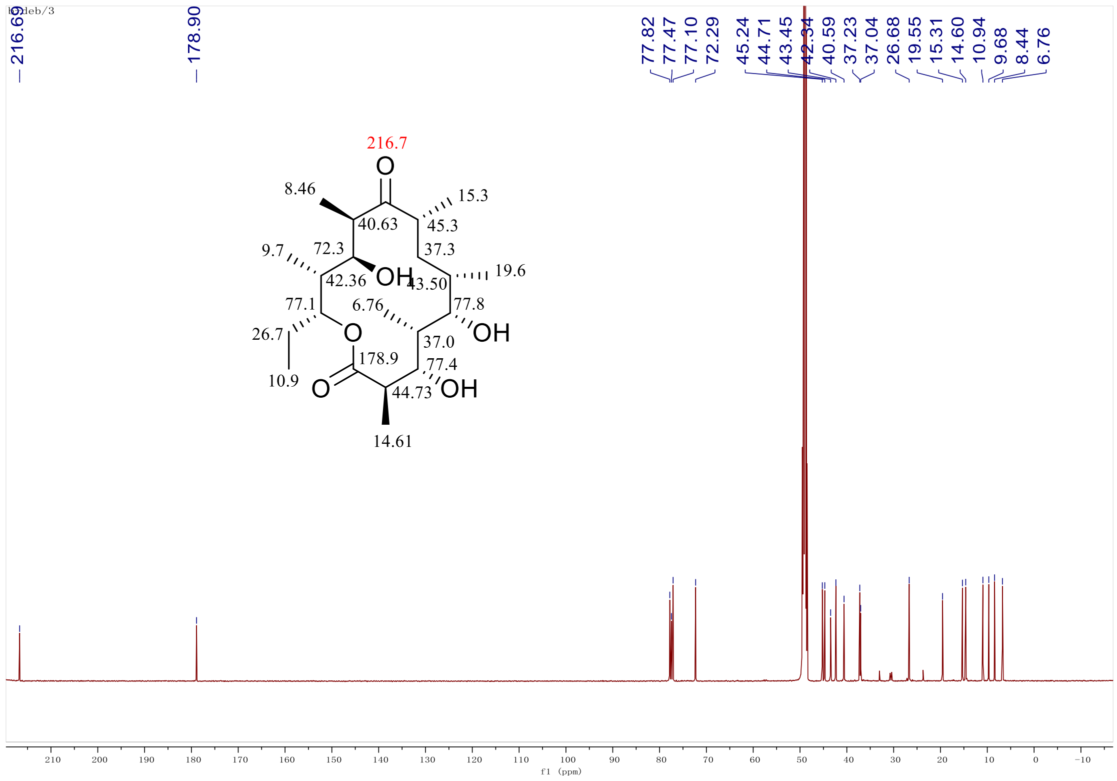

Fig. S2. ^{13}C NMR spectrum (125 MHz, CD_3OD) of 6-deoxyerythronolide B.

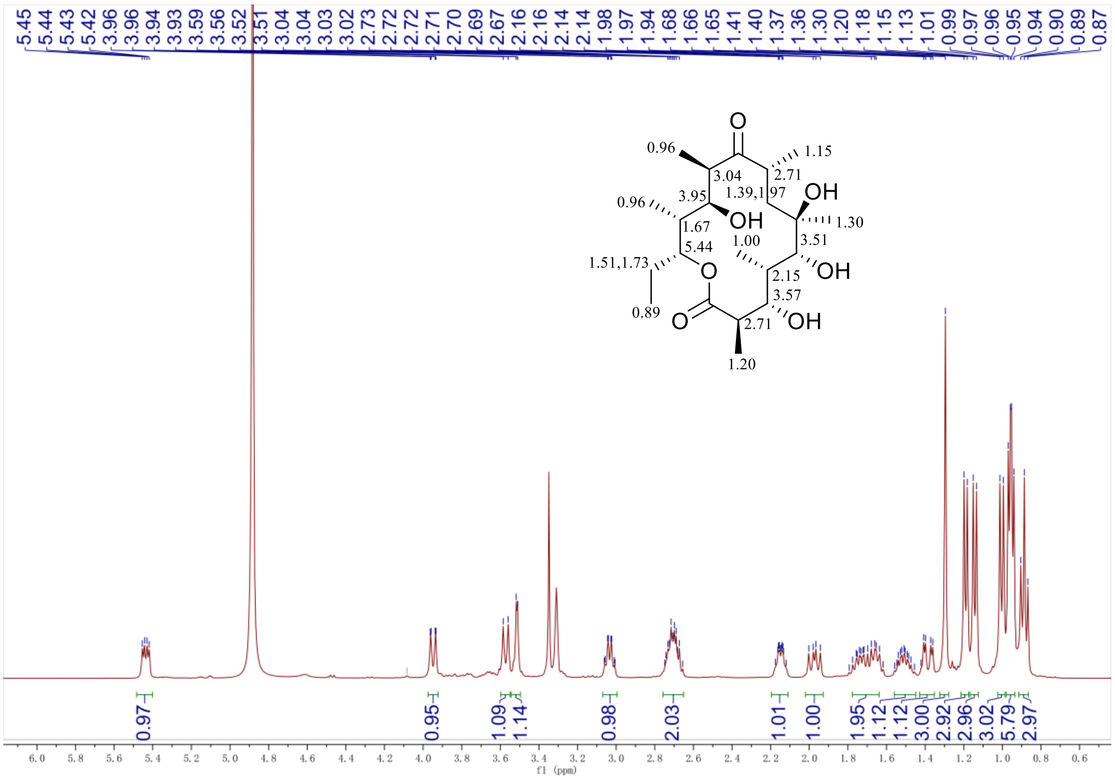

Fig. S3. ^{1}H NMR spectrum (500 MHz, CD_3OD) of erythronolide B.

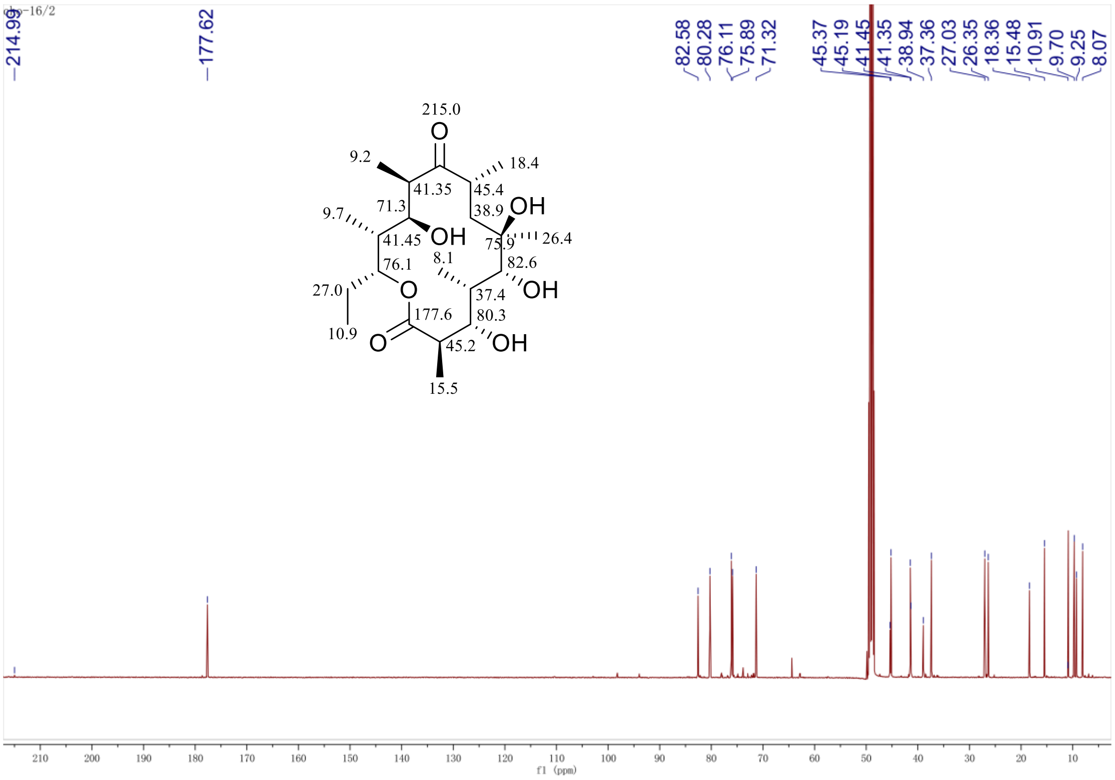

Fig. S4. ^{13}C NMR spectrum (125 MHz, CD_3OD) of erythronolide B.

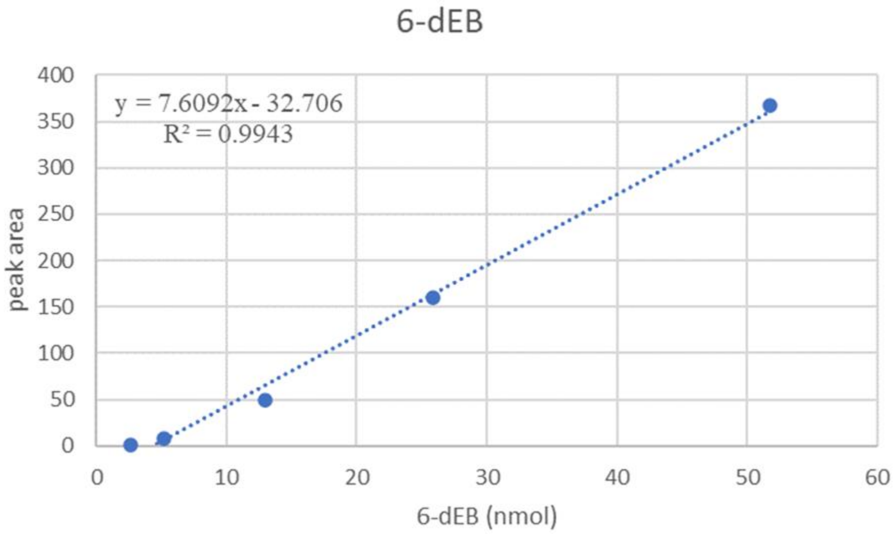

Fig. S5. The standard curve of 6-deoxyerythronolide B.

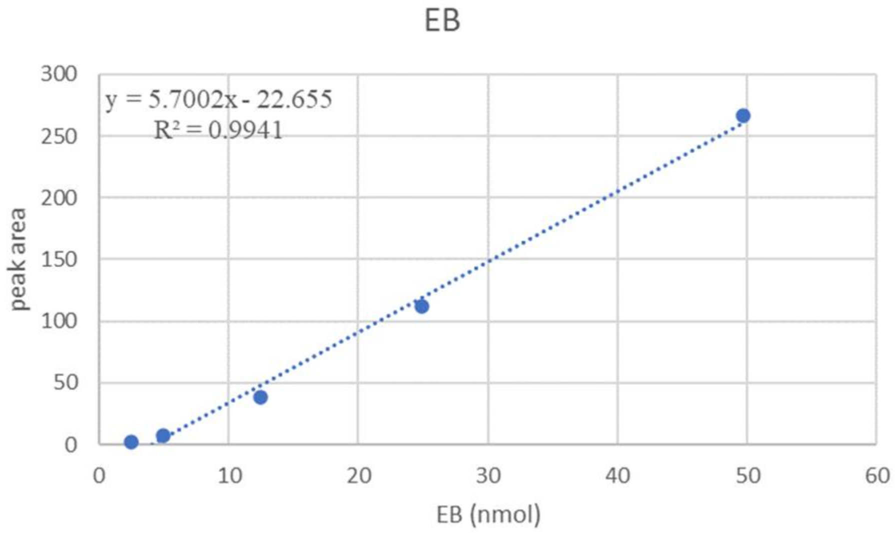

Fig. S6. The standard curve of erythronolide B.

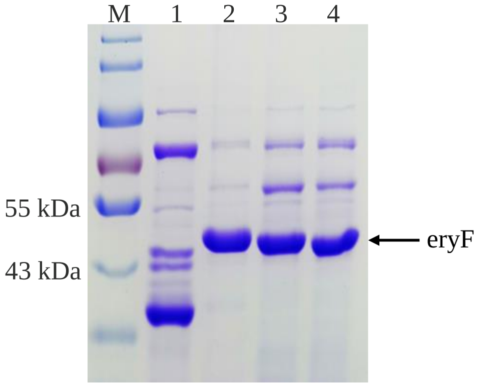

Fig. S7. SDS-PAGE examination of the expression of purified protein.

M represents protein marker; lane1, ferredoxin-NADP oxidoreductase from *Spinacia oleracea*; lane 2, SaEryF, P450EryF from *S. erythraea*; lane 3, AcEryF, P450EryF from *A. erythraea*; lane 4, AeEryF, P450EryF from *A. erythreum*.

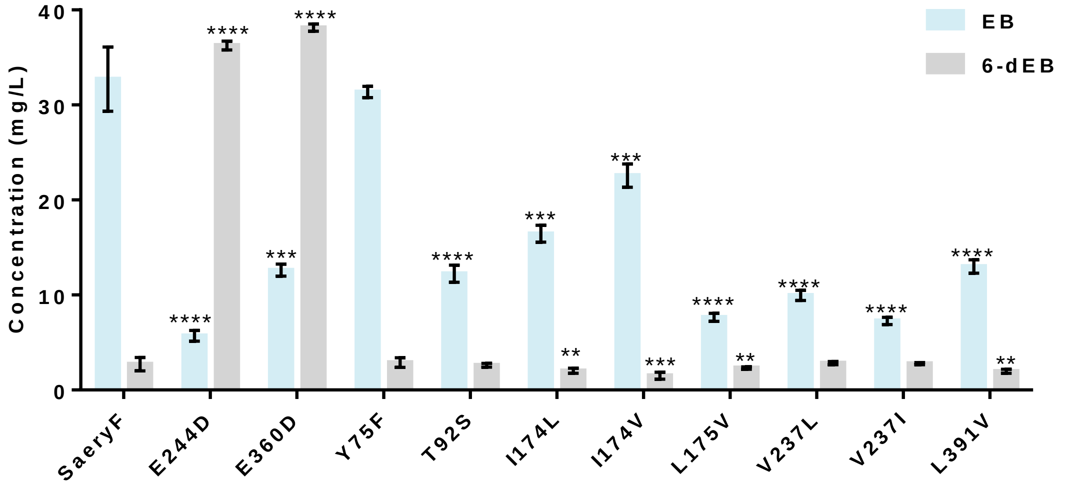

Fig. S8. The titer of EB and 6-dEB of mutants derived from substrate-binding pocket of SaEryF.

The data shown are means ± standard deviations calculated from triplicate individual experiments. Error bars show standard deviations. Statistical analysis was performed by a two-tailed Student's *t*-test. $*P < 0.05$, $**P < 0.01$, $***P < 0.001$, and $****P < 0.0001$ vs. the wild-type SaEryF.

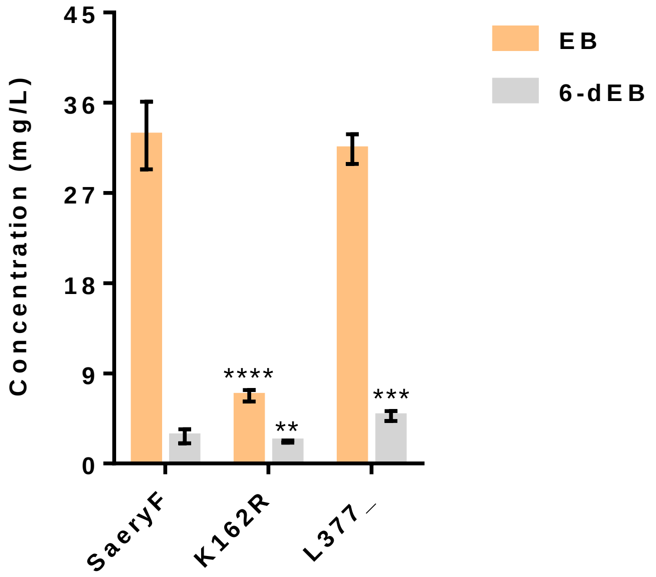

Fig. S9. The titer of EB and 6-dEB of mutants K162R and L377_.

L377_ means the deletion of amino acid Leu377. The data shown are means ± standard deviations calculated from triplicate individual experiments. Error bars show standard deviations. Statistical analysis was performed by a two-tailed Student's *t*-test. *$P < 0.05$, **$P < 0.01$, ***$P < 0.001$, and ****$P < 0.0001$ vs. the wild-type SaEryF.

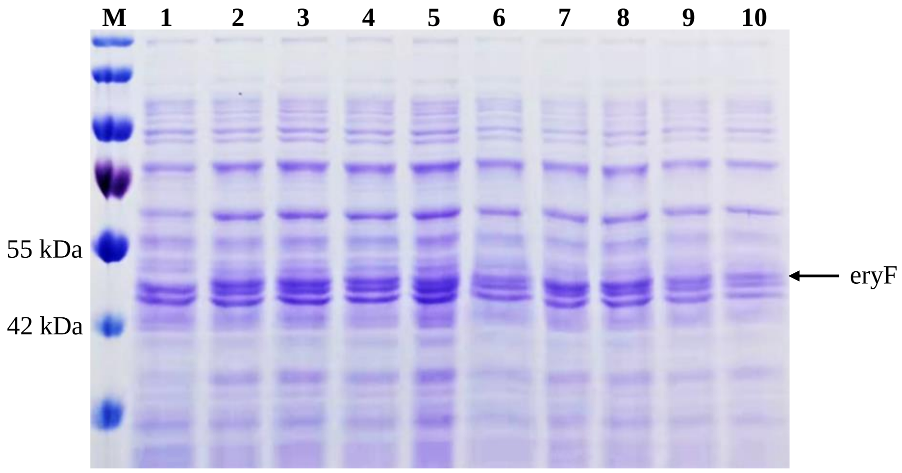

Fig. S10. SDS-PAGE examination of the expression of mutant protein.

M represents protein marker; lane 1, control plasmid pCDFDuet-1; lane 2, native SaEryF; lane 3, A74F; lane 4, N89Q; lane 5, L175I; lane 6, L391I; lane 7, K162G; lane 8, G165S; lane 9, I379V; lane 10, I379T.

Supplemental Tables

Table S1. Plasmids and strains for SaEryF mutation

Plasmid/strain	Description	Source
**Plasmid**		
pZF96	pZF84_derived, *SaEryF_A74F*	This study
pZF97	pZF84_derived, *SaEryF_Y75F*	This study
pZF98	pZF84_derived, *SaEryF_N89Q*	This study
pZF99	pZF84_derived, *SaEryF_T92S*	This study
pZF100	pZF84_derived, *SaEryF_I174L*	This study
pZF101	pZF84_derived, *SaEryF_I174V*	This study
pZF103	pZF84_derived, *SaEryF_L175I*	This study
pZF104	pZF84_derived, *SaEryF_L175V*	This study
pZF106	pZF84_derived, *SaEryF_V237L*	This study
pZF107	pZF84_derived, *SaEryF_V237I*	This study
pZF109	pZF84_derived, *SaEryF_L391V*	This study
pZF110	pZF84_derived, *SaEryF_L391I*	This study
pZF111	pZF84_derived, *SaEryF_E244D*	This study
pZF112	pZF84_derived, *SaEryF_E360D*	This study
pZF113	pZF84_derived, *SaEryF_K162R*	This study
pZF114	pZF84_derived, *SaEryF_K162G*	This study
pZF115	pZF84_derived, *SaEryF_Y163R*	This study
pZF116	pZF84_derived, *SaEryF_G165S*	This study
pZF117	pZF84_derived, *SaEryF_E166D*	This study
pZF118	pZF84_derived, *SaEryF_S376H*	This study
pZF119	pZF84_derived, *SaEryF_L377deletion*	This study
pZF120	pZF84_derived, *SaEryF_G378A*	This study
pZF121	pZF84_derived, *SaEryF_I379V*	This study
pZF122	pZF84_derived, *SaEryF_I379T*	This study
pZF123	pZF121_derived, *SaEryF_I379V_A74F*	This study
pZF124	pZF121_derived, *SaEryF_I379V_G165S*	This study
pZF125	pZF121_derived, *SaEryF_I379V_G165S_A74F*	This study
**Strain**		
sZF84	BAP1 carrying pBP130, pBP144, pZF84	This study
sZF85	BAP1 carrying pBP130, pBP144, pZF85	This study
sZF86	BAP1 carrying pBP130, pBP144, pZF86	This study
sZF96	BAP1 carrying pBP130, pBP144, pZF96	This study
sZF97	BAP1 carrying pBP130, pBP144, pZF97	This study
sZF98	BAP1 carrying pBP130, pBP144, pZF98	This study
sZF99	BAP1 carrying pBP130, pBP144, pZF99	This study
sZF100	BAP1 carrying pBP130, pBP144, pZF100	This study
sZF101	BAP1 carrying pBP130, pBP144, pZF101	This study
sZF103	BAP1 carrying pBP130, pBP144, pZF103	This study
sZF104	BAP1 carrying pBP130, pBP144, pZF104	This study

sZF106	BAP1 carrying pBP130, pBP144, pZF106	This study
sZF107	BAP1 carrying pBP130, pBP144, pZF107	This study
sZF109	BAP1 carrying pBP130, pBP144, pZF109	This study
sZF110	BAP1 carrying pBP130, pBP144, pZF110	This study
sZF111	BAP1 carrying pBP130, pBP144, pZF111	This study
sZF112	BAP1 carrying pBP130, pBP144, pZF112	This study
sZF113	BAP1 carrying pBP130, pBP144, pZF113	This study
sZF114	BAP1 carrying pBP130, pBP144, pZF114	This study
sZF115	BAP1 carrying pBP130, pBP144, pZF115	This study
sZF116	BAP1 carrying pBP130, pBP144, pZF116	This study
sZF117	BAP1 carrying pBP130, pBP144, pZF117	This study
sZF118	BAP1 carrying pBP130, pBP144, pZF118	This study
sZF119	BAP1 carrying pBP130, pBP144, pZF119	This study
sZF120	BAP1 carrying pBP130, pBP144, pZF120	This study
sZF121	BAP1 carrying pBP130, pBP144, pZF121	This study
sZF122	BAP1 carrying pBP130, pBP144, pZF122	This study
sZF123	BAP1 carrying pBP130, pBP144, pZF123	This study
sZF124	BAP1 carrying pBP130, pBP144, pZF124	This study
sZF125	BAP1 carrying pBP130, pBP144, pZF125	This study

Table S2. The primers for plasmids construction

Primer	Sequence（5’→3’）
Primer_84F	CATCACCATCATCACCACAGCCAGGATCCAATGACCACCGTGCCGGACCT
Primer_84R	TGCAGGCGCGCCGAGCTCGAATTCACTAGTTTAGCCATCCAGACGAACCG
Primer_85F	CATCACCATCATCACCACAGCCAGGATCCAATGACCACCGTGCCGGACCT
Primer_85R	TGCAGGCGCGCCGAGCTCGAATTCACTAGTTTAGCCCTCCAGACGAACCG
Primer_86F	CATCACCATCATCACCACAGCCAGGATCCAATGACCGCGCTGCCGGAAGT
Primer_86R	TGCAGGCGCGCCGAGCTCGAATTCACTAGTTTAGCCACCCAGACGAACCG
Primer224_F	CCCCTCTAGAAATAATTTTGTTTAACTTTAAGAAGG
Primer224_R	CCGAGCTCGAATTCACTAGTTTACATCATGCCGCCCATGCCAC
Primer_96F	GAGGTGGAATTCCCGTTTTATCTGGGCTTTCCGGAAGACG
Primer_96R	CGGGAATTCCACCTCAACACC
Primer_97F	GTGGAATTCCCGGCGTTTCTGGGCTTTCCGGAAGACG
Primer_97R	CGCCGGGAATTCCACCTCAAC
Primer_98F	AACTACTTCGCGACCCAGATGGGTACCAGCGATCCGC
Primer_98R	GGTCGCGAAGTAGTTACGAACG
Primer_99F	GCGACCAACATGGGTAGCAGCGATCCGCCGACCCAC
Primer_99R	ACCCATGTTGGTCGCGAAGTAG
Primer_100F	CGTTGGAGCAGCGAGCTGCTGGTGATGGACCCGGAACG
Primer_100R	CTCGCTGCTCCAACGGCCAAAT
Primer_101F	CGTTGGAGCAGCGAGGTGCTGGTGATGGACCCGGAACG
Primer_101R	CTCGCTGCTCCAACGGCCAAAT
Primer_103F	TGGAGCAGCGAGATCATCGTGATGGACCCGGAACGTG
Primer_103R	GATCTCGCTGCTCCAACGGCC
Primer_104F	TGGAGCAGCGAGATCGTGGTGATGGACCCGGAACGTG
Primer_104R	GATCTCGCTGCTCCAACGGCC
Primer_106F	ACCAGCATTGCGCTGCTGCTGCTGCTGGCGGGTTTTG
Primer_106R	CAGCGCAATGCTGGTCAGTTC
Primer_107F	ACCAGCATTGCGCTGATCCTGCTGCTGGCGGGTTTTG
Primer_107R	CAGCGCAATGCTGGTCAGTTC
Primer_109F	TGGCGTCGTAGCCTGGTGCTGCGTGGTATTGACCACC
Primer_109R	CAGGCTACGACGCCAAACCAC
Primer_110F	TGGCGTCGTAGCCTGATTCTGCGTGGTATTGACCACC
Primer_110R	CAGGCTACGACGCCAAACCAC
Primer_111F	CTGCTGGCGGGTTTTGATGCGAGCGTTAGCCTGATCG
Primer_111R	AAAACCCGCCAGCAGCAG
Primer_112F	CCGCTGGCGAAGCTGGATGGCGAAGTTGCGCTGCG
Primer_112R	CAGCTTCGCCAGCGGACG

Primer_113F	CTGGGTGTTGACGAGCGTTATCGTGGTGAATTTGGCCGTTGG
Primer_113R	CTCGTCAACACCCAGCAGTTCG
Primer_114F	CTGGGTGTTGACGAGGGTTATCGTGGTGAATTTGGCCGTTGG
Primer_114R	CTCGTCAACACCCAGCAGTTCG
Primer_115F	GGTGTTGACGAGAAGCGTCGTGGTGAATTTGGCCGTTG
Primer_115R	CTTCTCGTCAACACCCAGCAG
Primer_116F	GACGAGAAGTATCGTAGCGAATTTGGCCGTTGGAGCAGC
Primer_116R	ACGATACTTCTCGTCAACACCCAG
Primer_117F	GAGAAGTATCGTGGTGACTTTGGCCGTTGGAGCAGCG
Primer_117R	ACCACGATACTTCTCGTCAACACC
Primer_118F	CGTTTTCCGGCGCTGCACCTGGGTATTGATGCGGACGATG
Primer_118R	CAGCGCCGGAAAACGACC
Primer_119F	TTTTCCGGCGCTGAGCGGTATTGATGCGGACGATGTGG
Primer_119R	GCTCAGCGCCGGAAAACGAC
Primer_120F	CCGGCGCTGAGCCTGGCGATTGATGCGGACGATGTGGTTTG
Primer_120R	CAGGCTCAGCGCCGGAAAAC
Primer_121F	GCGCTGAGCCTGGGTGTGGATGCGGACGATGTGGTTTGG
Primer_121R	ACCCAGGCTCAGCGCCGG
Primer_122F	GCGCTGAGCCTGGGTACCGATGCGGACGATGTGGTTTGG

Table S3 Synthesized DNA sequences in this study

Gene	Sequence
EryF from *Saccharopolyspora erythraea*	ATGACCACCGTGCCGGACCTGGAGAGCGATAGCTTCCACGTTGA<br>CTGGTACCGTACCTATGCGGAGCTGCGTGAAACCGCGCCGGTGA<br>CCCCGGTTCGTTTTCTGGGTCAGGATGCGTGGCTGGTGACCGGTT<br>ATGATGAGGCGAAAGCGGCGCTGAGCGACCTGCGTCTGAGCAGC<br>GATCCGAAGAAAAAGTACCCGGGTGTTGAGGTGGAATTCCCGGC<br>GTATCTGGGCTTTCCGGAAGACGTTCGTAACTACTTCGCGACCAA<br>CATGGGTACCAGCGATCCGCCGACCCACACCCGTCTGCGTAAGC<br>TGGTTAGCCAGGAGTTTACCGTGCGTCGTGTTGAAGCGATGCGT<br>CCGCGTGTTGAGCAAATCACCGCGGAGCTGCTGGACGAAGTGGG<br>TGATAGCGGCGTGGTTGACATTGTTGATCGTTTCGCGCACCCGCT<br>GCCGATCAAAGTGATTTGCGAACTGCTGGGTGTTGACGAGAAGT<br>ATCGTGGTGAATTTGGCCGTTGGAGCAGCGAGATCCTGGTGATG<br>GACCCGGAACGTGCGGAACAGCGTGGTCAAGCGGCGCGTGAAG<br>TGGTTAACTTCATTCTGGACCTGGTGGAGCGTCGTCGTACCGAAC<br>CGGGTGACGATCTGCTGAGCGCGCTGATCCGTGTTCAGGACGAT<br>GACGATGGCCGTCTGAGCGCGGATGAACTGACCAGCATTGCGCT<br>GGTGCTGCTGCTGGCGGGTTTTGAGGCGAGCGTTAGCCTGATCG<br>GTATTGGCACCTACCTGCTGCTGACCCATCCGGACCAACTGGCG<br>CTGGTTCGTCGTGATCCGAGCGCGCTGCCGAACGCGGTTGAGGA<br>AATCCTGCGTTATATTGCTCCGCCGGAAACCACCACCCGTTTTGC<br>GGCGGAGGAAGTGGAGATCGGTGGCGTTGCGATTCCGCAGTACA<br>GCACCGTGCTGGTTGCGAACGGTGCGGCGAACCGTGACCCGAAA<br>CAATTCCCGGACCCGCACCGTTTTGACGTGACCCGTGATACCCGT<br>GGCCACCTGAGCTTCGGTCAAGGCATCCACTTTTGCATGGGTCGT<br>CCGCTGGCGAAGCTGGAGGGCGAAGTTGCGCTGCGTGCGCTGTT<br>CGGTCGTTTTCCGGCGCTGAGCCTGGGTATTGATGCGGACGATGT<br>GGTTTGGCGTCGTAGCCTGCTGCTGCGTGGTATTGACCACCTGCC<br>GGTTCGTCTGGATGGCTAA
EryF from *Actinopolyspora erythraea*	ATGACCACCGTGCCGGACCTGGAAAGCGATAGCTTCCACGTTGA<br>CTGGTACCGTACCTATGCGGAGCTGCGTGAAACCAGCCCGGTGA<br>CCAGCGTTCGTTTTCTGGGTCAGGACGCGTGGCTGATTACCGGCT<br>ATGATGAAGCGAAAGCGGCGCTGAACGACCTGCGTCTGAGCAGC<br>GATCCGAAGAAAAAGTACCCGGGTGTTGAGGTGGAATTCCCGGC<br>GTATCTGGGCTTTCCGGAGGACGTGCGTAACTACTTCGCGAACA<br>ACATGGGTACCAGCGATCCGCCGACCCACACCCGTCTGCGTAAA<br>CTGGTTAGCCAGGAGTTTACCGTGCGTCGTGTTGAAGCGATGCG<br>TCCGCGTGTTGAGCGTATCACCAGCGACCTGCTGGATCAACTGG<br>GTGATAGCGGTGAAGGCGACGTGGTTGATCGTTTCGCGCACCCG<br>CTGCCGATCAAAGTGATTTGCGAGCTGCTGGGCGTTGACGAACG<br>TTACCGTGGTGATTTTGGCCGTTGGAGCAGCGAAATTCTGGTGAT<br>GGCGCCGGAACGTGCGGAAGCGCGTGGTGAGGCGGCGCGTGAA<br>ATCGTTAACTTCATTCTGGAGCTGATCGAACGTCGTCGTACCGAG

	CCGGGTGATGATCTGCTGAGCGGTCTGATCCGTGTGCAGAACGA<br>CGATGCGGACCGTCTGAGCGCGGATGAACTGGCGAGCGTGAGCC<br>TGGTTCTGCTGCTGGCGGGCTTTGAGGCGAGCGTTAGCCTGATCG<br>GTATTGGCACCTATCTGCTGCTGACCCATCCGGAGCAACTGGCG<br>CTGGTGCGTCGTGACCCGAGCGCGTGGCCGAACGCGGTTGAGGA<br>AATTCTGCGTTGCATCACCCCGCCGGAAACCACCACCCGTTTCGC<br>GACCGAGGAACTGGAGATTGGTGGCGTGACCATCCCGCGTTATA<br>GCACCGTGCTGGTTGCGGGTGGCGCGGCGAACCGTGACCCGAAA<br>CAGTTCCCGAACCCGGACCGTTTTGATGTGACCCGTGATACCCGT<br>GGTCACCTGGCGTTCGGTCAAGGCATTCACTTTTGCATGGGTCGT<br>CCGCTGGCGAAGCTGGAGGGCGAAGTTGCGCTGCGTGCGCTGTT<br>CGAACGTTTTCCGGACCTGAGCCTGGGCGTGGATGCGGACGATG<br>TTCTGTGGCGTCGTAGCCTGCTGCTGCGTGGTATCGACCACCTGC<br>CGGTTCGTCTGGAGGGCTAA
EryF from *Aeromicrobium erythreum*	ATGACCGCGCTGCCGGAAGTGCCGGACCTGGATAGCGACGCGTT<br>CCACGTTGACTGGTACGATACCTATGCGCAACTGCGTGAGCGTC<br>GTCCGGTGACCCCGGTTCGTTTCTTTGGTCAGGACGCGTGGCTGG<br>TGACCGGCTATGAACAAGCGCGTACCGCGCTGACCGACCTGCGT<br>CTGAGCAGCGATCCGAAGGCGCAATACCCGGATGTGGACGTTGA<br>TTTCCCGGCGTATCTGGGTTTTAGCGACCGTGCGAAGCACTACTT<br>CGTTAACAACATGGGTACCAGCGATCCGCCGAGCCACACCCGTC<br>TGCGTAAACTGGTGGCGCGTGAGTTTACCGCGCGTCGTGTTATG<br>GCGATGCGTCCGCGTGTGCAGCAAATCGTTGACGGTCTGCTGGA<br>TACGATGGCGGAAACCCCGGACGCGGATGTTGTTGCGAGCTTCG<br>CGCACCCGCTGCCGATCCAAGTGATTTGCGAGCTGCTGGGTGTT<br>GAGGAAGGCCGTCGTAGCGACTTTGGTCGTTGGAGCGCGGAAAT<br>CCTGATTATGGACCCGGAGCGTGCGGAAGCGCGTGGTGCGGCGG<br>CGGAGGAAGTGGTTGACTTCATGCTGGATCTGGTGGAGCGTCGT<br>CGTGCGGCGCCGGGTGATGATCTGCTGAGCGCGCTGATTCAGGC<br>GCGTGACGTTGATGAGGCGCGTCTGAGCCAAGACGAACTGGTGA<br>GCGTTGCGCTGGTGCTGCTGCTGGCGGGCTATGAGGCGAGCGTT<br>AGCCTGATCGGTATTGGCAGCTACCTGATGCTGCGTCACCGTGA<br>ACAGCTGGATGTGCTGAAAGCGGACCCGACCCTGTGGCCGAACG<br>CGGTGGAGGAAGTTCTGCGTCTGTATGCTCCGCCGGAGACCACC<br>ACCCGTTTTGCGGCGCAAGACGTGGAAATCGATGGTGTTAGCAT<br>TCCGGCGTATAGCATGGTGCTGGTTGCGGGTGCTGCGGCGAACC<br>GTGACCCGGCGCGTTTCCCGGACCCGGATCGTTTTGACGTGCGTC<br>GTGATACCAAAGGTCACCTGACCTTCGGTCACGGCATCCACCAC<br>TGCCTGGGTCGTCCGCTGGCGATGCTGGAGGGCGAAGTTGCGCT<br>GCGTAGCCTGTTCGAACGTTTTCCGGATGTGCATGCGACCGACCT<br>GGATGCGGTTGCGTTTCGTCGTAGCCTGCTGCTGCGTGGCATTGA<br>TAGCCTGCCGGTTCGTCTGGGTGGCTAA

Ferredoxin-NADP reductase (FNR) from *Spinacia oleracea*	ATGACCACCGCCGTGACCGCCGCCGTGAGCTTTCCGAGCACCAAAACCACCAGTCTGAGTGCCCGTAGTAGTAGTGTGATTAGTCCGGATAAAATTAGCTATAAAAAGGTTCCGCTGTATTATCGTAATGTTAGCGCCACCGGCAAAATGGGTCCGATTCGTGCCCAGATTGCCAGTGATGTGGAAGCCCCGCCGCCGGCACCGGCAAAAGTTGAAAAACATAGTAAAAAGATGGAGGAAGGTATTACCGTTAATAAGTTTAAACCGAAAACCCCGTATGTTGGTCGCTGCCTGCTGAATACCAAAATTACCGGCGATGATGCCCCGGGCGAAACCTGGCACATGGTGTTTAGCCATGAAGGTGAAATTCCGTATCGTGAAGGCCAGAGCGTGGGTGTGATTCCGGATGGCGAAGATAAAAATGGTAAACCGCATAAACTGCGCCTGTATAGTATTGCAAGCAGTGCCCTGGGTGACTTTGGTGACGCAAAAAGCGTGAGTCTGTGTGTGAAACGTCTGATCTATACCAATGATGCAGGTGAAACCATTAAGGGTGTGTGTAGTAATTTTCTGTGTGATCTGAAACCGGGCGCCGAAGTTAAACTGACCGGCCCGGTGGGCAAAGAAATGCTGATGCCGAAAGATCCGAATGCAACCATTATTATGCTGGGCACCGGCACCGGTATTGCCCCGTTTCGTAGCTTTCTGTGGAAAATGTTTTTCGAAAAACACGATGATTACAAGTTCAATGGTCTGGCCTGGCTGTTTCTGGGTGTTCCGACCAGTAGTAGTCTGCTGTATAAAGAAGAATTTGAAAAGATGAAGGAGAAGGCCCCGGATAATTTTCGCCTGGATTTTGCCGTTAGTCGTGAACAGACCAATGAAAAAGGTGAAAAAATGTATATCCAGACCCGCATGGCACAGTATGCCGTGGAACTGTGGGAAATGCTGAAAAAAGATAATACCTATGTGTACATGTGCGGCCTGAAAGGTATGGAAAAAGGCATTGATGATATTATGGTGAGCCTGGCCGCAGCCGAAGGTATTGATTGGATTGAATATAAACGCCAGCTGAAAAAAGCCGAACAGTGGAATGTTGAAGTGTATTAA
